# Catastrophic failure due to aggressive metallosis 4 years after hip resurfacing in a woman in her forties—a case report

**DOI:** 10.3109/17453674.2010.487246

**Published:** 2010-05-21

**Authors:** Thord von Schewelov, Lennart Sanzén

**Affiliations:** Department of Orthopedics, Lund University, Malmö University Hospital, MalmöSweden

## Introduction

**Figure 1.**
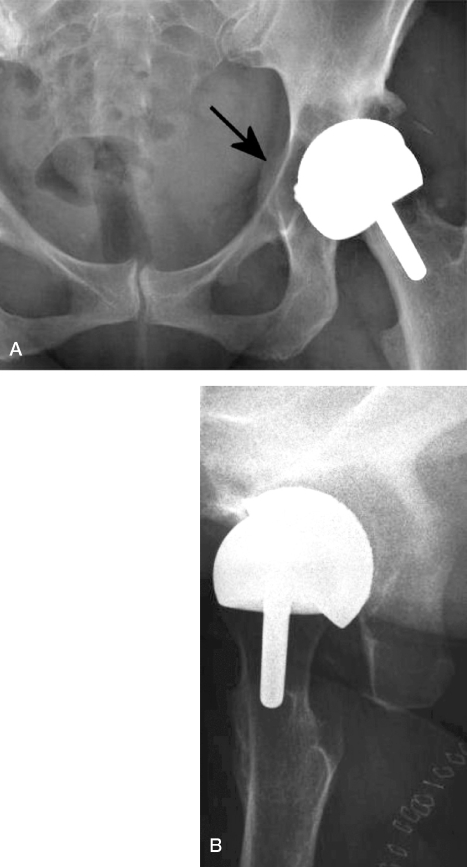
A. View of the lower pelvis before revision. Note the marked abduction angle of the cup and the destruction around both implants with protrusion of metallosis into the pelvis (arrow). B. Marked anteversion of the acetabular component.

In March 2005, a 42-year-old healthy woman underwent a hip resurfacing procedure with a Birmingham metal-on-metal hip resurfacing implant (Smith and Nephew Orthopaedics, Warwick, United Kingdom) because of osteoarthritis secondary to mild hip dysplasia. After 4 years, she reported the onset of mild discomfort and instability in her hip and 6 months later she was referred to our unit because of radiographically visible aggressive periprosthetic osteolysis (Figure 1) and progressive pain. ESR and CRP were normal. The BHR implant is made of cobalt and chromium, and the levels of these metals in the blood were grossly elevated (Table).

**Table:** Metal concentrations, μg/L, (95% CI) in whole blood analyzed with inductively coupled plasma mass spectrometry (ICP-SMS) by ALS Scandinavia AB, Luleå, Sweden

	Before revision	6 weeks postoperatively	4 months postoperatively	Reference ^**a**^
Cobalt	92 (74–110)	11 (9–13)	2 (1.4–2.0)	0.09 (< 0.02–0.26)
Chromium	59 (47–72)	16 (13–20)	8 (7–10)	0.51 (< 0.4–1.2)
Molybdenum	1.8 (1.5–2)	0.4 (0.3–0.4)	0.3 (0.2–0.3)	0.9 (0.21–5.41)

^**a**^ Reference values according to Rodushkin et al. (1999), median range.

**Figure 2.**
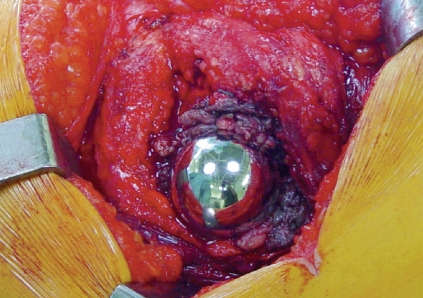
Metallosis surrounding the implant and cervical neck.

**Figure 3.**
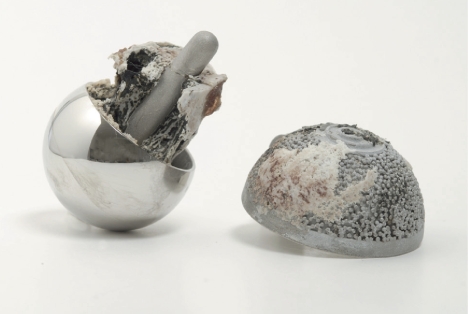
The revised implant.

**Figure 4.**
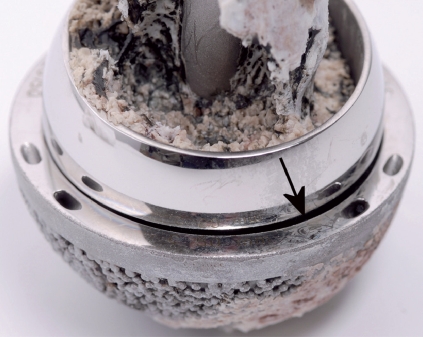
Macroscopic wear (arrow).

At surgery, we found a massive aggressive metallosis in and around the joint (Figure 2). The metallosis had eroded half the cervical neck (Figure 3). The acetabular component was still fixed in approximately 55 degrees of abduction and about 45 degrees of anteversion (Figure 1) with metallosis, a black-stained granulation tissue present all around the rim. After detachment from the only remaining area of intact bone, about 2 cm in diameter postero-laterally (Figure 3), we found that the rest of the acetabulum was heavily eroded. A thin unicortical shell of the anterior column remained. A thin bone bridge posterio-medially remained of the posterior column; the rest was destroyed. An elliptical 2.5 × 3 cm defect in the medial wall was demarcated by fibrous tissue. There were no signs of infection. When the two prosthetic components were put together, an obvious macroscopic asymmetry of the articulation was observed, representing excessive wear (Figure 4).

**Figure 5.**
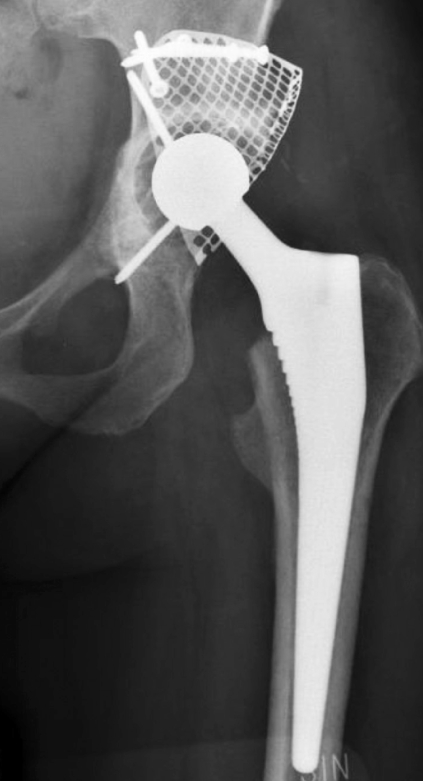
Postoperatively.

We reconstructed the acetabulum with structural allografts and impaction bone grafting. A large rim mesh substituted the posterior and cranial defects. A cemented cross-linked Marathon polyethylene cup and a Corail stem were then implanted (Figure 5).

## Discussion

Hip resurfacing techniques were introduced in the 1970s with the aim of minimizing bone resection, wear, and risk of dislocation, but the method was discarded because of a high failure rate. With the evolution of implant manufacturing methods, hip resurfacing was reintroduced in the 1990s and promising short-term results have been reported (Steffen et al. 2008). However, there are an increasing number of reports of serious complications with this type of implant. An increased risk of revision has also been reported, e.g. from the Australian hip registry (http://www.dmac.adelaide.edu.au/aoanjrr).

A review of the biological reactions around metal-on-metal implants has been published by Mabilleau et al. (2008). The complications include periprosthetic soft tissue destruction, osteolysis, pseudotumors, and infiltrates of lymphocytes and plasma cells. These infiltrates are thought to represent an immunological response to metal debris (Mahendra et al. 2009). The term aseptic lymphocytic vasculitis-associated lesion (ALVAL) has been introduced (Pandit et al. 2008). High cobalt (Co), chromium (Cr), and molybdenum (Mo) levels in the blood from patients with metal-on-metal implants have been reported by several authors. Apart from the direct adverse periprosthetic effects of these particles, the 10- to 1,000-fold increase in blood Co, Cr, and Mo concentrations may have systemic effects (Mabilleau et al. 2008, Hart et al. 2009). Whether these are of clinical importance is still unclear.

There is an increased risk of revision related to the head size of the surface replacement especially if the acetabular component is placed in excessive abduction and/or anteversion (http://www.dmac.adelaide.edu.au/aoanjrr), as in our case, leading to an increased edge loading of the bearing surface and causing increased production of metal wear particles (Ollivere et al. 2009). An increased risk of revision was also observed in the Australian registry in 2009 for all women, and for men over 60 years of age. Grammatopoulos et al. (2009) found that although one of the alleged advantages of hip resurfacing should be an easier revision, revision of these implants for inflammatory pseudotumor or metallosis unfortunately has a poor outcome.

Hip resurfacing with a metal-on-metal articulation may prove to be successful in well-chosen patients and with meticulous implant positioning, but there have been sufficient reports of severe early complications not seen with any other kinds of hip prostheses to suggest that great caution should be exercised when using this type of implant.
